# A Sport Monitoring System Based on the Optimized Adaptive Fuzzy PID Control Algorithm in OneNet Internet of Things and Cloud Platform

**DOI:** 10.1155/2022/8234066

**Published:** 2022-04-21

**Authors:** Di Zhang, Hyun Joo Min

**Affiliations:** ^1^Department of Sports, Qinghai University, Xining 810016, China; ^2^College of Art and Physical Education, Gangneung-Wonju National University, Gangneung 210600, Republic of Korea

## Abstract

Aiming at some problems existing in the existing sports monitoring system, based on the joint action of OneNet Internet of Things (IoT) and cloud platform, an optimized adaptive fuzzy PID control algorithm is adopted to monitor and analyze sports. Finally, the accuracy of the optimization model is verified through the comparison of different models, and the algorithm is used to predict and analyze sports. The research shows that (1) as the index calculation of cloud platform shows, with the increase of iteration time, the change curve of relevant indexes can be divided into four different stages, namely, rapid fluctuation stage, slow decline stage, slow fluctuation stage, and rapid decline stage. (2) The conventional calculation method (CPID) cannot well describe the change rule of the test data in the early stage of settlement. The fuzzy adaptive calculation method (NPID) also exposes some errors in the fitting and description of the test curve in the calculation process, while the improved adaptive calculation method (GPID) can describe the change characteristics and rules of the test curve well for different stages. (3) Compared with the original model, the optimization model can better describe the first and second stages of index change, indicating the accuracy of the optimization model. And the algorithm can be used to predict and analyze the changes of indicators and sports monitoring better, and the analysis results can provide relevant guidance for sports monitoring. This optimization scheme provides basis and theoretical support for the application of OneNet IoT and cloud platform.

## 1. Introduction

The Internet of Things (IoT) is a network that uses information sensing devices to connect materials and the Internet for information exchange and communication, so as to realize intelligent identification, positioning, tracking, monitoring, and management. Compared to the traditional models, the IoT has the characteristics of strong penetration, great driving effect, and good comprehensive benefits. It is another promoter of the development of information industry after computer, Internet, and mobile communication.

 The application and development of the IoT are conducive to promoting the transformation of production, life, and social management to intelligent, refined, and networked one and greatly improving the level of social management and public services. It will foster a large number of new technologies, new products, new applications, and new models, upgrade traditional industries and transform the mode of economic development, and become a growth point for future economic development. We can understand the IoT from the following two aspects: first, we should make it clear that IoT is the extension and expansion of the Internet, and its core and foundation are still the Internet; secondly, we should fully realize that the users of IoT include people and objects, so that the IoT really realizes the exchange of information between people and objects. As a new research method, the IoT is playing an important role in various aspects: in order to improve the application range of the currently, commonly used multipath transmission organization method to the IoT devices. Morawski et al. [1] proposed a system tuning method for energy consumption optimization architecture module. A new scheduler and path manager is designed by using formal optimization method. To further verify the accuracy of the optimization model, relevant data were used for verification and analysis, and the results show that the optimization model based on the IoT theory can further improve the accuracy and adaptability of the structure. The application of the IoT in environmental mapping and other fields is mainly manifested as monitoring data. To solve the problem that GPS cannot accurately locate and navigate in the process of indoor operation of the IoT, based on the relevant theories of the IoT, Chhikara et al. [2] processed the image by using the correlation algorithm of deep neural network. It is worth noting that the genetic algorithm of cloud computing was adopted to realize the optimization analysis of many parameters of CNN. In order to verify the accuracy of the model, relevant experimental data were used to verify the model results, and the results show that the optimized IoT model can be well applied in related fields. To better explore the application of the IoT in related fields, Ravi et al. [3] adopted a corresponding network detection system of the IoT based on two-layer deep learning framework in view of the problems existing in the IoT. The framework model has high scalability and correctness on hardware server because of the cloud computing platform algorithm. By using the monitoring data set to evaluate the proposed optimization framework, the research shows that the optimization model can not only describe smart city construction well, but also can optimize and guide the related problems in urban construction by predicting the relevant indicators.

The above studies are mainly analyzed from the application of IoT, mainly including structural module optimization, image processing based on deep learning, and optimization of network monitoring system. The IoT can not only be optimized for the model or system, but also be combined with the cloud platform to analyze and optimize the structure, so that the optimized results can better meet relevant needs. Therefore, researches on cloud platform computing are attracting more and more scholars' attention: to select appropriate methods to effectively monitor and record the workflow of related products. Zhang et al. [4] developed a public service cloud platform based on batch identification and record preservation based on cloud platform and OneNet calculation method. Compared with the traditional methods, the efficiency of cloud platform data acquisition and monitoring using this optimization method is improved by about 20%. Intelligent teaching plays an important role in practical teaching. In order to better optimize relevant processes and evaluation indicators of intelligent teaching, Liu et al. [5] proposed an accurate learning resource teaching model based on cloud platform and deep neural network representation based on security and decentralization of blockchain. Based on the cloud platform of smart learning, the relevant indicators and characteristics of smart teaching demand based on big data technology are analyzed by using relevant optimization methods to construct the relevant detail framework of smart teaching. The construction method of cloud platform can effectively promote the allocation and sharing of learning resources and provide more accurate and personalized learning support and services for teachers and students. The cloud platform can put forward specific opinions and predictions according to the specific characteristics of the structure, but there are also many problems in the operation of the cloud platform. In view of many problems existing in the cloud platform, Ge et al. [6] can accurately locate the original data by optimizing the process and details in the monitoring data. Then, on the basis of the data, the optimized cloud platform is used to sort out and analyze the data, put forward relevant suggestions, and predict the problems and applications existing in the data. To verify the accuracy of the model, the relevant data of the pollution degree of a certain area is used for analysis. The results show that cloud platform can better describe the law of pollution value and then give the relevant prediction results. Huang et al. [7] made a sensor component with long distance and good sensitivity based on relevant intelligent composite materials under cloud computing platform in order to better meet the relevant requirements of users for strain products with both long distance measurement capability and good sensitivity. The use of IoT cloud platform computing under the sensor can detect and capture a wide range of human activities, and it also suitable for robot movement and vehicle safety collision real-time monitoring. It is worth noting that the component structure successfully sends relevant real-time monitoring data signals to the main station of the platform through the big data cloud platform, then carries out accurate analysis and research on the relevant situation, and verifies the accuracy of the model through relevant test data and monitoring results.

In addition to cloud platform, artificial intelligence (AI) and IoT enabled monitoring platforms have been widely applied to various fields. For example, to optimize the monitoring system of autonomous guided vehicles, an integrated IoT architecture based on deep neural network and modified linear element is proposed. The optimization model can provide reliable and safe online monitoring [8]. To diagnose and optimize power transformers, a new integrated IoT architecture and deep learning online monitoring method is applied to power transformers. This approach can improve the effectiveness of IoT architectures [9]. In order to expand the monitoring application of IoT and AI in medical services, Kishor et al. [10] proposed a machine learning-based healthcare model to early and accurately predict the different diseases with the help of IoT. Mansour et al. [11] proposed a new disease diagnosis model of intelligent medical system based on the fusion of artificial intelligence and Internet of Things, and the experiment showed that the model had high diagnostic accuracy for heart disease and diabetes. Aydın et al. [12] used IoT devices and sensors to obtain a wide range of information from irrigation areas and normalized the data to build corresponding AI models. The model can assist the operator in making a series of decisions about the irrigated area. Artificial intelligence has great potential in the field of collecting data for analysis and realizing agricultural automation intelligence by using wireless sensor network technology. Vijayakumar and Balakrishnan [13] found a correct system architecture by testing various machine learning algorithms. Studies show that these algorithms can produce 95% accuracy compared with other systems. Due to the simplicity of the structure algorithm of the existing sports monitoring system, the calculation results have a certain one-sidedness, leading to a large deviation between the results and the actual situation, which cannot describe the relevant characteristics of students in sports, so that the calculation results cannot provide a theoretical basis for relevant policies. The existing sports monitoring system cannot better describe the relevant characteristics of students in sports, and to better describe and optimize the sports monitoring system, this paper adopts OneNet IoT. Based on cloud computing platform, the adaptive PID control algorithm and theory in monitoring control principle are used to modify the original model. Thus, the modified adaptive fuzzy PID control algorithm and system are obtained. After further optimization, the sports monitoring system based on OneNet IoT and cloud platform is obtained. The model is verified by using relevant data. The results show that, compared with the traditional model, the optimized model can well describe the related indicators of sports, providing theoretical support for the application and development of OneNet IoT and cloud platform. By using the optimized sports monitoring system, the Internet of Things platform can collect and process the data transmitted through the network, relying on the Internet of Things platform to achieve the management and automation of intelligent objects. The sports monitoring system based on OneNet IoT and cloud platform can make the solution of sports more cost effective. It also provides better security and data mobility, which in turn enhances student collaboration. In addition, the IoT and cloud platform also provides greater flexibility to provide further developed capabilities to meet different conditions and environments.

## 2. OneNet IoT and Cloud Platform

Cloud computing is a computing mode based on Internet management and services. This computing method can conveniently access shared resources, which mainly include networks, servers, storage, applications, and services. By using this method, the exchange and communication between IoT and cloud platform can be well promoted. To solve the various problems existing in the various computing services on the cloud platform, Yun et al. [14] proposed a new ASLR technology based on cloud computing platform, which provides a method of memory to optimize the design and analysis of the model without interrupting the process execution. In addition, in order to better calculate the model under OneNet IoT platform accurately, through accurate analysis and research on the transmission data, the data can be optimized through relevant calculation, so as to provide computing support for the precise positioning of the model. In recent years, cloud computing, as a new research method, is often adopted to improve the computing and data storage capacity of power system control center. However, there are also some problems in cloud computing platform. Ma et al. [15] proposed a new control center framework based on cloud platform under the influence of cloud computing methods. The optimized cloud computing system can improve the accuracy of power grid intelligent load data collection and carry out relevant analysis and classification of characteristic data according to different input parameters, so as to meet the requirements of different types of data. On the basis of the above model, a data feature extraction method combined with PID adaptive fuzzy model is proposed. The precision of this method can be upgraded through the training of recurrent neural network (RNN). To verify the accuracy and correctness of the model, through the analysis and classification of monitoring data in a certain area, and compared with other conventional methods, the research results show that the optimization model can meet the requirements of data precision and can further improve the adaptability of the model. To study the accuracy and rules of structural control under different cloud computing platforms, Xiong et al. [16] adopted a unique computing method and cloud platform to store different types of data and encrypt the original data using the optimization algorithm of advanced encryption standard. The results show that the encrypted data using the optimized algorithm can further improve the security, and the accuracy of the relevant structures and frameworks based on cloud platform computing can be further improved.

**Figure 1:**
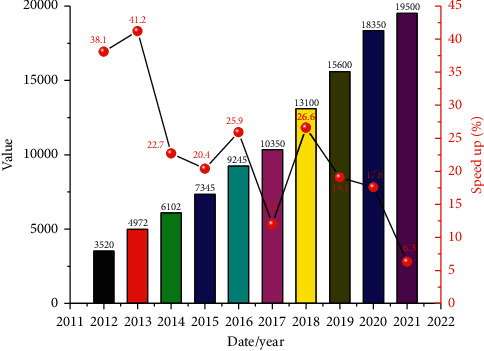
Scale and growth rate of the IoT in the past decade.

IoT, as a new monitoring and use platform [17, 18], is further increasing with the rapid development of economy. The scale change in the past decade is shown in Figure 1, as can be seen from the figure: (1) with the growth of time, the industrial scale of IoT shows a linear increase trend, increasing from only 352 billion in 2012 to nearly 2 billion in 2021, an increase of about 5.5 times. (2) As can be seen from the annual growth rate curve, with the increase of time, the growth rate gradually decreased from the initial close to 40% to 6.3%, a decrease of about 84%.

### 2.1. Cloud Platform

**Figure 2:**
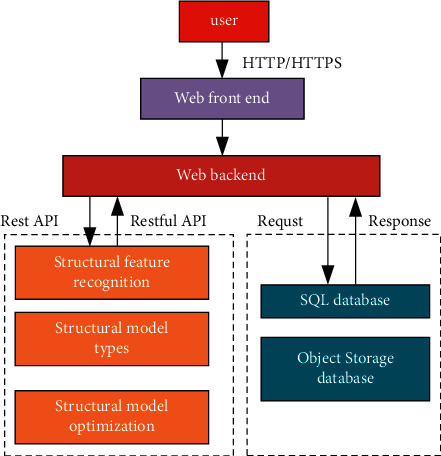
Overall design of cloud platform.

Through the above research, we can see that cloud platform under the effect of IoT is growing rapidly. Cloud platform, as a new research method and means, is slowly changing people's lifestyle and providing research ideas and methods to promote the interconnection between OneNet and cloud platform. The architectural core of cloud computing is cloud service, which consists of software Service Layer (SaaS), platform service layer (PaaS), and infrastructure layer (IaaS) [19]. The overall system design of the cloud platform is shown in Figure 2 through cloud computing, network function virtualization, multiheterogeneous data database storage, and other key technologies.

**Figure 3:**
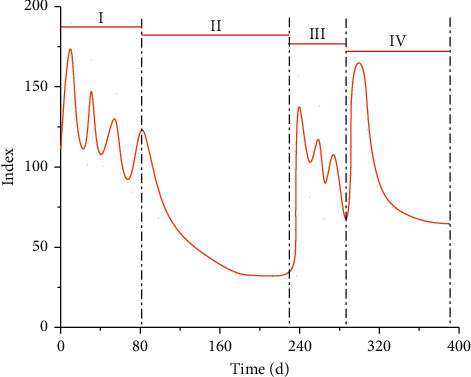
Changes of related indicators of cloud platform computing.

As can be seen from the overall design drawing of the cloud platform, differences in structural feature recognition, structural model types, and structural model optimization can lead to differences in cloud platform calculation and optimization results, resulting in different degrees of errors in corresponding models. To better explore the change rule of related indicators based on cloud platform, the change diagram of related indicators of cloud platform computing is drawn, as shown in Figure 3.

As can be seen from Figure 3, with the increase of iteration time, the change curves of relevant indicators can be divided into four different stages: rapid fluctuation stage, slow decline stage, slow fluctuation stage, and rapid decline stage. First of all, the curve shows rapid fluctuation. With the acceleration of fluctuation, the index value of peak point gradually decreases. When the second stage is reached, the curve shows a nonlinear slow decline, and the curve is approximately a logarithmic function trend. When the index reaches the lowest point, the curve enters the third stage, in which the fluctuation amplitude of the curve tends to decline, making the index decrease slowly. With the increase of time, the curve enters the fourth stage, and the slope of the corresponding curve declines rapidly first, then slowly, and finally approximates to 0.

### 2.2. IoT

Through the correlation analysis of the IoT and cloud platform, it can be seen that, according to the law of diminishing marginal utility, the utility function is assumed to be a smooth and monotonous convex function. The function is described by exponential function, and the change rule of the IoT and cloud platform is characterized by studying the quantitative relationship of different resources. It is worth explaining that introducing two variables to modify the corresponding utility function makes the obtained function more reliable. The relevant convex utility function is [20, 21]

(1) $$\begin{array}{c} U\left(x_{1},x_{2}\right)=\frac{x_{1}^{1-\delta_{1}}}{1-\delta_{1}}+\frac{x_{2}^{1-\delta_{2}}}{1-\delta_{2}}, \end{array}$$

where *x*_1_ and *x*_2_ represent the different resources represented in the function, respectively; *δ*_1_ and *δ*_2_ represent the shape of the utility function.

The corresponding utility function is as follows:

(2) $$\begin{array}{c} U\left(x_{1},x_{2}\right)=\frac{x_{1}^{1-\delta_{1}}}{1-\delta_{1}}+\frac{x_{2}^{1-\delta_{2}}}{1-\delta_{2}},\delta_{i}>0,\delta_{i}\neq 1. \end{array}$$

Let *c*_1_ and *c*_2_ be the corresponding resource costs, respectively, and then the total cost can be expressed as

(3) $$\begin{array}{c} C\left(x_{1},x_{2}\right)=c_{1}x_{1}+c_{1}x_{1}+c_{0}, \end{array}$$

where *c*_0_ is the initial resource cost of the function.

The maximum value of the utility function can be expressed as

(4) $$\begin{array}{c} U^{\max}=U\left(b_{1},b_{2}\right)=\frac{b_{1}^{1-\delta_{1}}}{1-\delta_{1}}+\frac{b_{2}^{1-\delta_{2}}}{1-\delta_{2}}, \end{array}$$

where *b*_1_ and *b*_2_ are relevant parameters of the model.

The corresponding maximum cost is expressed as

(5) $$\begin{array}{c} C^{\max}=C\left(b_{1},b_{2}\right)=c_{1}b_{1}+c_{1}b_{1}+c_{0}. \end{array}$$

Therefore, the cost performance ratio of Internet cloud platform is defined as follows:

(6) $$\begin{array}{c} \theta =\left(X_{1},X_{2},\cdots ,X_{m}\right)=\frac{\sum_{i=1}^{m}U_{i}\left(X_{i}\right)}{\sum_{i=1}^{m}C_{i}\left(X_{i}\right)+c_{0}}, \end{array}$$

where *X*_*i*_ is the model input value.

The global optimality conditions are as follows [22, 23]:

(7) $$\begin{array}{c} L\left(\theta ,a\right)=\left\{\left(X_{1},X_{2},\cdots ,X_{m}\right)\in D|\theta \left(X_{1},X_{2},\cdots ,X_{m}\right)\geq a,a\in R\right\}. \end{array}$$

**Figure 4:**
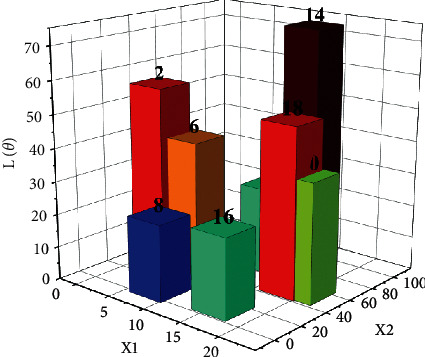
Computing results in the IoT and cloud platform.

Through the above calculation and analysis, the optimal conditions based on the cloud platform of the IoT can be obtained, and the computing diagram can be obtained through the limitation of the optimal conditions, as shown in Figure 4.

### 2.3. Framework for OneNet

**Figure 5:**
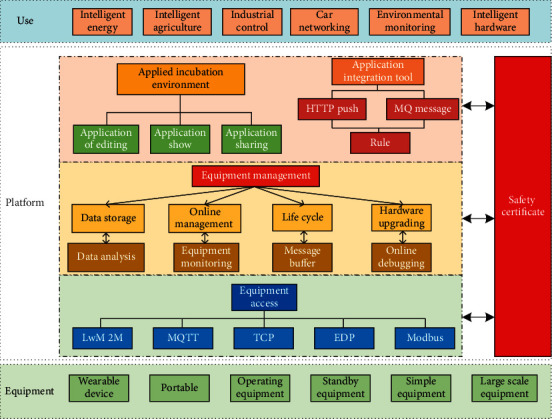
Basic frame diagram of OneNet.

The cloud platform of OneNet has obvious leading advantages, with multiprotocol configuration, API, online debugging, and many other functions, which can make it easy to complete the connection between devices. The basic framework of OneNet can be divided into application levels, platform levels, and device levels, as shown in Figure 5. The main application fields of OneNet include energy, industry, agriculture, intelligent operation, and system detection. The corresponding platform of OneNet can be divided into application incubation environment platform and application integration tool platform. These two platforms are mainly operated by corresponding applications and managed by device access.

The application link and the device link are auxiliary functions. These two links mainly conduct security authentication on the platform level, so that the application incubation and application integration in the platform can give full play to the functions of device management and device access. The intelligent analysis and processing of input data can be achieved, and the quantitative characterization of relevant indicators can be obtained, which can guide and forecast the development of relevant industries. The comparison of computing methods of relevant cloud platforms is shown in Table 1.

The constraint infinitive corresponding to the cloud platform based on OneNet is shown as follows [24, 25]:

(8) $$\begin{array}{c} f_{jk}\left(X\right)=c_{jk}-\sum_{i=1}^{m}x_{ijk}\geq 0,\;j\in J,k\in \Gamma ,\\ f_{ijk}\left(X\right)=x_{ijk}\geq 0,\;i\in I,j\in J,k\in \Gamma . \end{array}$$

The classical Lagrange solution method is as follows:

(9) $$\begin{array}{c} L\left(X,\lambda \right)=-U\left(X\right)-\sum_{s=1}^{N}\lambda_{s}f_{s}\left(X\right). \end{array}$$

Exponential Lagrange function is as follows:

(10) $$\begin{array}{c} F_{M}\left(X,\lambda \right)=-U\left(X\right)-M^{-1}\sum_{s=1}^{N}\lambda_{s}\left(1-e^{-Mf_{s}\left(X\right)}\right). \end{array}$$

Define the set as follows [26, 27]:

(11) $$\begin{array}{c} E^{M}\left(\varepsilon ,\delta \right)=E_{1}^{M}\left(\varepsilon ,\delta \right)\times \cdots \times E_{N}^{M}\left(\varepsilon ,\delta \right),\\ D\left(\varepsilon ,\delta \right)=\left\{\left(\lambda ,M\right)|\lambda \in E^{M}\left(\varepsilon ,\delta \right),M\geq M_{0}\right\}. \end{array}$$

**Figure 6:**
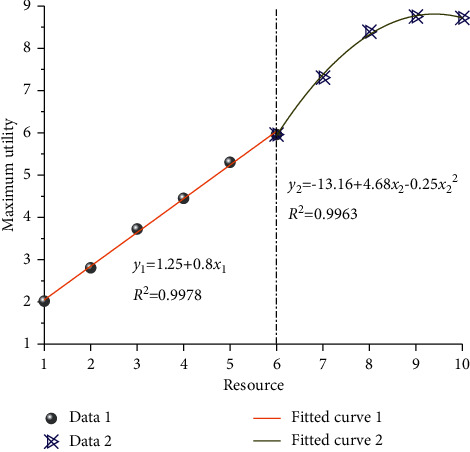
Variation curves of resources and maximum effect.

Through the above theoretical analysis, the variation rules of different resources and maximum effect are obtained, and the maximum effect curves under different resources are drawn, as shown in Figure 6. As can be seen from the figure above, with the gradual increase of resources, the maximum utility of OneNet cloud platform presents a trend of gradual increase, which can be divided into two parts, namely, linear change stage and nonlinear change stage. (1) When resources are low, the linear feature between maximum utility and resources is particularly obvious. After linear function fitting, it is found that the corresponding fitting coefficient is about 0.9978. For a given function resource, the corresponding maximum utility can be obtained through the corresponding relationship, and the corresponding calculation amount can be further reduced by adopting this method. (2) With the gradual increase of resources, the nonlinear relationship between maximum utility and resources gradually shows up. The curve gradually tends to be stable, and the slope of the corresponding curve gradually decreases until it reaches 0. It is found that the corresponding fitting coefficient is about 0.9963 by using the quadratic function relation to fit the curve, which indicates that when the resource is large, the relation conforms to the quadratic function distribution. The critical value of linear and nonlinear relationship is 6.

## 3. Monitoring and Control Principle

### 3.1. PID Control Algorithm

**Figure 7:**
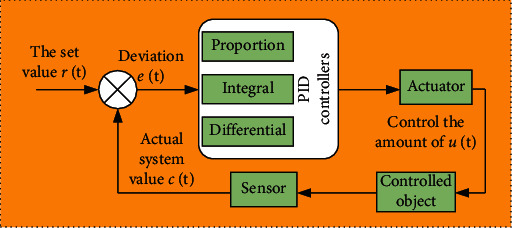
PID control schematic diagram.

PID is a control algorithm integrating proportion, integral, and differential [28, 29]. Also known as proportional integral differential control, the control schematic diagram of PID algorithm is obtained through analysis, as shown in Figure 7. Firstly, the set value is input into the corresponding system, and the corresponding deviation and error are obtained through calculation. Then, the error is imported into the PID controller, and the error is divided into three aspects. Finally, the corresponding actual system value is obtained through the calculation and operation of the actuator, control object, and sensor.

E(*t*) is the system control error, and the calculation formula is as follows [30, 31]:

(12) $$\begin{array}{c} e\left(t\right)=r\left(t\right)-c\left(t\right), \end{array}$$

where the *r*(*t*) is the expected value of the variable; *c*(*t*) is the actual value of the system.

Its control law is as follows:

(13) $$\begin{array}{c} u\left(t\right)=K_{p}\left[e\left(t\right)+\frac{1}{T_{i}}\int_{0}^{t}e\left(t\right)+T_{D}\frac{de\left(t\right)}{\text{d}t}\right]. \end{array}$$

The corresponding conversion function is shown as follows:

(14) $$\begin{array}{c} G\left(s\right)=\frac{U\left(S\right)}{E\left(S\right)}=K_{p}\left(1+\frac{1}{T_{i}S}+T_{D}S\right). \end{array}$$

The corresponding general expression is

(15) $$\begin{array}{c} u\left(t\right)=k_{p}e\left(t\right)+k_{i}\int e\left(t\right)\text{d}t+k_{d}\frac{\text{d}e\left(t\right)}{\text{d}t}. \end{array}$$

The corresponding discrete expression can be written as [32, 33]

(16) $$\begin{array}{c} u\left(k\right)=k_{p}e\left(k\right)+k_{i}\sum e\left(k\right)+k_{d}\left[e\left(k\right)-e\left(k-1\right)\right], \end{array}$$

where the *u*(*k*) is the control output; *K*_*p*_ is the corresponding proportional growth coefficient; *K*_*i*_ is the coefficient of integral accumulative term; *K*_*d*_ is the coefficient of differential term; *T*_*i*_ is the corresponding integral time constant; *T*_*d*_ is the differential time constant.

**Figure 8:**
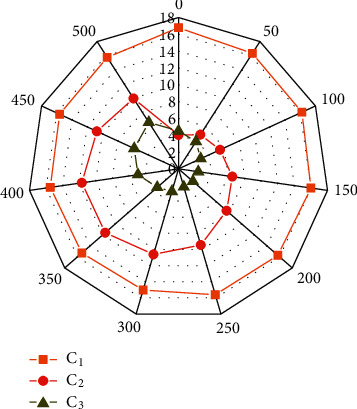
Radar diagram of different parameters.

Different parameters in the PID have a great influence on the model results. Therefore, to explore the influence degree and change rule of different parameters, the parameter change diagram under different iteration steps is drawn, as shown in Figure 8. As can be seen from the figure above, the changes of model parameters under different iteration steps are slightly different: (1) it can be seen from C_1_ that as the number of iteration steps increases, the curve changes approximately horizontally and fluctuates around 17. This parameter has good stability. (2) It can be seen from C_2_ that when the number of iteration steps is small, the variation range of parameters is large. As the number of iteration steps increases, the curve corresponding to parameters gradually tends to be gentle, and the variation range decreases. (3) As can be seen from C_3_, the overall change of parameters is relatively small, the stability is poor, and the overall impact on the model is relatively small.

### 3.2. Adaptive Fuzzy PID Control Algorithm

The adaptive fuzzy PID control algorithm adopted in this system establishes control rules through the fuzzy relationship between the system temperature deviation *e*, deviation change rate *ec* and three related parameters *K*_*p*_, *K*_*i*_ and *K*_*d*_ of PID control algorithm [34, 35].

(17) $$\begin{array}{c} K_{p}=K_{p}^{\prime }+\Delta K_{p}^{\prime },\\ K_{i}=K_{i}^{\prime }+\Delta K_{i},\\ K_{d}=K_{d}^{\prime }+\Delta K_{d}. \end{array}$$

where the △*K*_*p*_ is the correction of the proportional coefficient; △*K*_*p*_ is the correction of the integral coefficient; △*K*_*p*_ is the correction of the differential coefficient.

**Figure 9:**
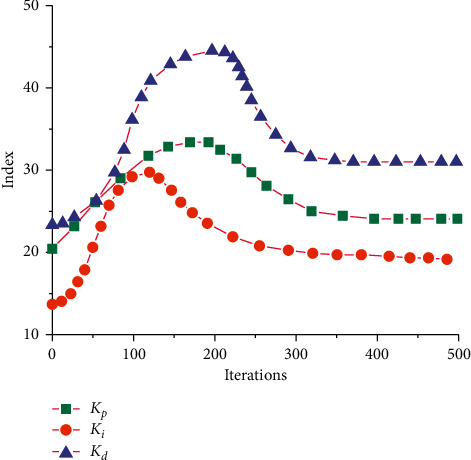
Iterative changes of different parameters.

To explore the influence of different parameters on the adaptive fuzzy control algorithm and analyze the influence of relevant parameters on the control rules, the change curves of relevant parameters under different iterations were drawn, as shown in Figure 9. As can be seen from the figure above, different curves rise rapidly at first and then decline slowly as the number of iterations increases until the curves gradually tend to be flat and reach a stable state. Different parameters have different effects on the curve. *K*_*i*_ reaches the maximum value when the number of iteration steps is about 110. The parameter has the least influence on the model. When the number of iteration steps of *K*_*p*_ is about 200, the curve reaches the maximum value of the corresponding index, indicating that this parameter has a great influence on the model. *K*_*d*_ reaches the maximum value when the number of iteration steps is about 220, indicating that this parameter has the greatest influence on the model.

Based on the above analysis, it can be seen that *K*_*p*_ has the greatest influence on the model algorithm. On this basis, we will explore the change rule of function assignment of system control variables and obtain the assignment data of related functions through calculation. The summary statistics are shown in Table 2.

### 3.3. Modified Adaptive Fuzzy PID Control Algorithm

**Figure 10:**
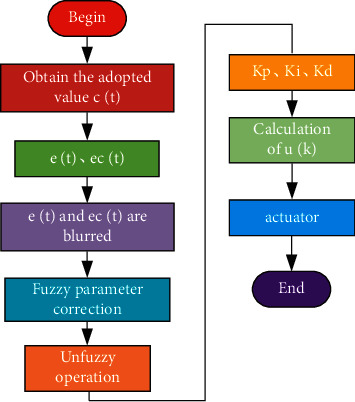
Flow chart of improved PID control algorithm.

The basic idea of the modified adaptive fuzzy algorithm is to set the threshold for the deviation value e(t). When the deviation is less than a certain value, the integral term is accumulated, and when the deviation exceeds a certain value, the accumulation stops [36]. The specific improved PID control algorithm flow is shown in Figure 10 [37, 38].

The improved formula is shown as follows [39]:

(18) $$\begin{array}{c} u\left(k\right)=k_{p}e\left(k\right)+Xk_{i}\sum e\left(k\right)+k_{d}\left[e\left(k\right)-e\left(k-1\right)\right],\\ X=\left\{\begin{array}{cc} 1, & e\left(k\right)<a\\ 0, & e\left(k\right)>a \end{array}.\right. \end{array}$$

The transfer function of the system is as follows [40]:

(19) $$\begin{array}{c} G\left(s\right)=\frac{K\times e^{-rs}}{Ts+1}, \end{array}$$

where the *K* is static increment; *T* is a time constant; *R* is the lag time related parameter, and the selected related parameters are as follows: *K* is 0.8, *T* is 80, and *r* is 8. Substitute the above relevant parameters into the corresponding transfer function:

(20) $$\begin{array}{c} G\left(s\right)=\frac{0.8\times e^{-8s}}{80\times s+1}. \end{array}$$

The relevant parameters of PID were calculated by the relevant parameter mediation method, as shown below [41, 42]:

(21) $$\begin{array}{c} \frac{K_{p}=0.8Tr}{K},\\ \frac{K_{i}=K_{p}}{T},\\ K_{d}=K_{p}\times T_{d}. \end{array}$$

**Figure 11:**
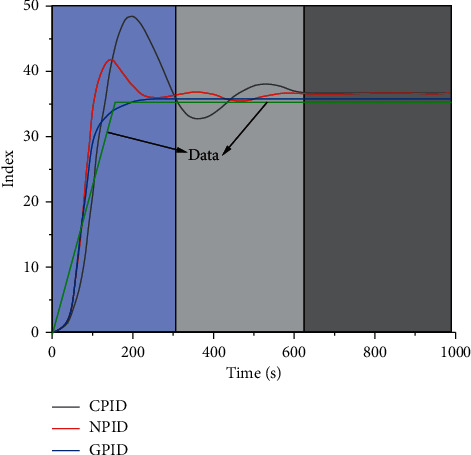
Comparison of different schemes.

Different model schemes have different influences on the calculation results. According to the relevant calculation results and steps, three main calculation schemes are obtained. In order to better analyze the influence of different schemes on the model indicators, the indicator change diagrams under different schemes are drawn, as shown in Figure 11.

It can be seen from the figure above that the numerical value can be divided into two parts as a whole: the first part is an approximate linear growth stage with a constant slope. When it reaches the highest point, the curve enters the second part. The second part is also a linear stage; the slope is approximately 0, and the curve is approximately horizontal. From different calculation methods, the curve can be divided into three stages, which are fast fluctuation stage, slow fluctuation stage, and stable stage, respectively. (1) In the first stage, CPID generally shows a change rule of rapid rise first and then rapidly declines, and the amplitude of fluctuation, maximum and minimum values, deviate from the test data to a large extent. In the second stage, the curve fluctuates slowly up and down, and the vibration range is near the test data, approximately showing a sinusoidal fluctuation change law. In the third stage, the curve is approximately stable. (2) It can be seen from the NPID curve that, in the first stage, its change pattern is basically the same as that of CPID, but the change range and scope are slightly decreased. In the stage of slow fluctuation, we can see that the curve gradually becomes stable with the increase of time, and the variation range is smaller and smaller, and it is closer to the experimental value. In the stable phase, the curve also maintains a constant level change. (3) From the perspective of GPID, the first stage shows a trend of rapid rise, and the corresponding curve slope rises slowly at first and then gradually declines. The maximum value of the curve is basically consistent with the test data, and in the second and third stages, the curve basically coincides with the test data.

From the above analysis, it can be seen that the conventional calculation method (CPID) cannot well describe the change rule of test data in the early stage of settlement. Fuzzy adaptive calculation method (NPID) also exposed some errors in the fitting and description of the test curve in the calculation process and could not accurately describe the changes of the test curve. As an improved adaptive calculation method (GPID), it can describe and characterize the characteristics and rules of the test curve in the first, second, and third stages; especially the characteristic points of the test curve can be described more accurately.

## 4. Optimization of Sports Monitoring System

### 4.1. Sports Introduction

As an important link in the growth of students, school sport is an indispensable part of the overall development of “morality, intelligence, physique, Beauty, and labor.” However, there are some typical problems in the existing sports monitoring system, which are shown as follows:

1. Outdated monitoring system: the existing monitoring system mainly adopts the single-factor method to detect and analyze students' related sports indicators, and the monitoring results are relatively single. The results obtained from the analysis are far from the actual students' sports indicators, failing to provide good guidance for students' sports.
2. The monitoring and evaluation indicators are not clear: the existing evaluation indicators fail to keep up with the times and the requirements of education in the new era, and the evaluation of students is relatively single, failing to reflect the principle of being above students. It makes students' enthusiasm for sports gradually disappear, and only relying on compulsory implementation cannot promote the healthy development of students. Relevant measures should be adopted to further clarify the evaluation indicators of students.
3. Lack of supervision mechanism: the current supervision mainly focuses on the self-supervision of PE teachers, which is easy to cause the lack of a certain degree of formality and logic in PE classes, which cannot play a positive role in the physical and mental development of students. Various monitoring methods should be adopted to supervise related monitoring systems; for example, (a) campus monitoring can be the main, using the relevant mechanisms of campus sports for strict supervision and guidance; (b) the use of relevant laws and regulations is enacted by the government to supervise sports with governmental supervision as the legal and institutional support; (c) under the principle of self-supervision, teachers and students should act as users and advisors of the sports monitoring system, as well as supervisors; (d) social supervision, as a supplement to the sports monitoring system, provides positive suggestions and suggestions on relevant deficiencies. Regarding a detection system for sports in the process of implementing a series of related problems, this article adopts the related technologies of IoT cloud platform to modify existing sports detection system and optimization. The optimized sports system can better reflect the performance and change of students in sports and carry out targeted guidance.

### 4.2. Optimize Content and Results

**Figure 12:**
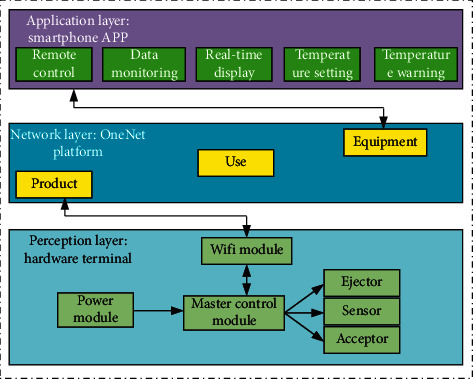
Overall system architecture diagram.

The sports monitoring system under the cloud platform is optimized, processed, and analyzed by using the concept of OneNet and the IoT. The overall framework of the relevant system is shown in Figure 12 [43]. According to different functions, it can be divided into application layer, network layer, and perception layer. On the one hand, smart phones and computers are used to monitor and analyze relevant data in real time. Through the analysis of the existing sports indicators, if the situation is different from the optimization standard, timely measures should be adopted to solve the problem through relevant early warning and remote control. On the other hand, in the power module, generator generation, sensing, and receiving are mainly carried out through the main control module, and relevant information is transmitted to the module.

Through the correlation analysis of different sports, the statistical table of sports system evaluation indicators is obtained, as shown in Table 3.

### 4.3. Application of Sports Monitoring System

**Figure 13:**
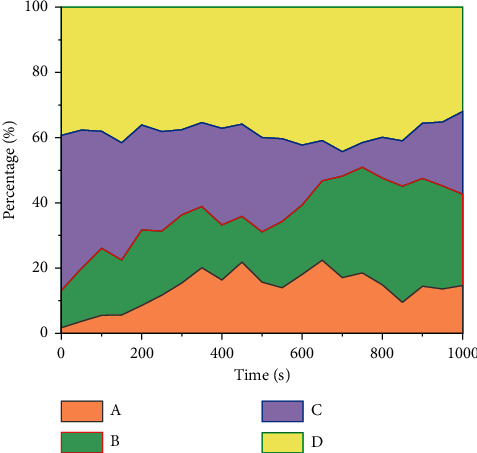
Accumulation area diagram under different classification indexes.

To better explore the application of different classification indexes in the sports monitoring system based on OneNet IoT and cloud platform, the accumulation area diagram under different classification indexes is drawn, as shown in Figure 13.

As can be seen from the figure above, with the increase of calculation steps and calculation time, the variation trends of different classification indexes are different: (1) as can be seen from indicator A, with the increase of time, the proportion of indicator A shows a trend of rapid rise at first and then fluctuation. After reaching the 850 s, the proportion of index A suddenly changes from A slow decline to an upward trend. (2) As can be seen from indicator B, with the increase of calculation time, the proportion of indicator B keeps an approximately constant trend before 600 s, and the proportion increases very little. When the time exceeds 600 s, the curve begins to increase significantly, indicating that 600 s is the midpoint of index B's change. (3) From the perspective of indicator C, the proportion of indicator C decreases rapidly until the time is 700 s, and the minimum value of indicator C appears at 700 s. After 700 s, the proportion of indicator C begins to increase slowly. (4) From the perspective of indicator D, the proportion of indicator D is approximately constant, accounting for about 20% of the total, indicating that indicator D is the influencing factor with the highest proportion.

**Figure 14:**
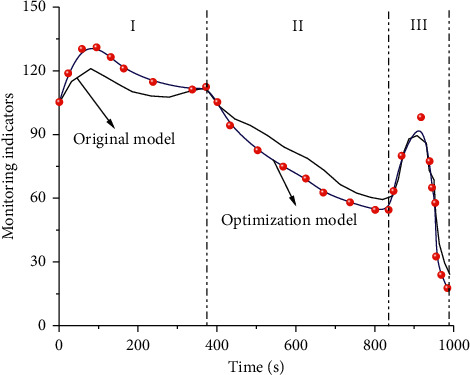
Comparison of different models.

By adopting the above analysis methods and theories and based on the computing concept of OneNet IoT and cloud platform, the comparison diagram between the theoretical optimization model and the original model under different computing time is obtained by adopting the optimized calculation method, as shown in Figure 14. It can be seen from the above figure that the original model cannot well describe the change trend of sports monitoring indicators, while the optimized model can well represent the change trend of sports indicators. According to the morphology, it can be divided into three stages: (1) the first stage is a fluctuation stage, during which the sports monitoring indicators show a trend of slowly increasing first and then gradually decreasing with the increase of calculation time. (2) The second stage is a linear decline stage. In this stage, the index curve shows an approximate linear decline rule with a change time of about 500 s. (3) The third stage is the rapid fluctuation stage. Although the time in this stage is short, the value change of the index has a very obvious jump. It first increases rapidly, then reaches the highest point in this stage, and then decreases rapidly. From a quantitative point of view, the original model failed to give a good description of the first and second stages, and the description error was large. The original model could only give a good description of the third stage. The optimized model can not only describe the first and second stages of sports indicators, but also represent the third stage of indicators. It indicates that the model optimized by OneNet IoT has high adaptability and correctness.

**Figure 15:**
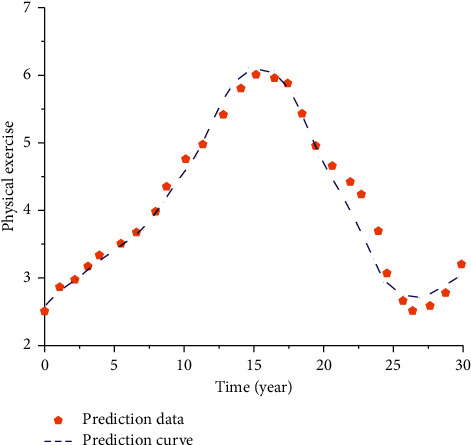
Sports prediction chart.

It can be seen from the above analysis that the sports monitoring system using the optimized OneNet IoT cloud platform can well represent the change process and change rules of sports. To better predict related indicators of sports, curves of sports changes at different times are drawn, as shown in Figure 15.

As can be seen from the figure above, this model can well describe the changing rules of sports. Overall, sports have shown a rapid development in the past 15 years. After a long time of development, sports may face a certain period of decline 15 years later, which needs to adopt some measures for prevention and control, so that sports can continue to develop. Meanwhile, according to the fitting degree of prediction data and prediction curve, it can be seen that the curve fitting before 15 years is better, while the curve fitting after 15 years is generally lower than the fitted value. It indicates that, with the improvement of the research time, the accuracy of the corresponding fitting curve decreases gradually. This requires us to optimize and analyze the monitoring system based on OneNet IoT and cloud platform according to the changes in the research content, so as to better meet the prediction of test data.

## 5. Conclusion

Based on the computing theory of OneNet IoT and cloud platform, this paper adopts the modified PID control algorithm to optimize and analyze the monitoring system of sports. Relevant conclusions are as follows:

1. The increase of resources makes the maximum utility of OneNet cloud platform gradually increase. When the resources are low, the linear characteristics between maximum utility and resources are obvious. With the gradual increase of resources, the nonlinear relationship between maximum utility and resources gradually shows up, and the curve tends to be stable.
2. The influences of different parameters in the PID calculation algorithm on the model results are shown as follows: the corresponding curve of C_1_ changes approximately horizontally as the number of iterative steps increases, indicating that the parameter has good stability. When the number of iteration steps of C_2_ is small, the variation range of parameters is large. As the number of iteration steps increases, the curve corresponding to parameters gradually tends to be gentle. The overall change of C_3_ parameter is relatively small, and the overall impact on the model is relatively small.
3. Both the original model and the optimized model can better describe the overall shape of the curve, which can be divided into fluctuation stage, linear decline stage, and rapid fluctuation stage. From the quantitative point of view, the original model can only describe the third stage well. And the precision of the description result is poor, while the optimized model can well represent each stage of the index, and the precision of description is good. The results show that the model can be used to analyze the monitoring index. In addition, it can be seen from the study that, with the increase of time, the fitting accuracy of the corresponding model decreases gradually. It shows that we should optimize the model according to the specific situation of the test data, so that the optimized model can better reflect the actual situation of the monitoring system.

For the Internet of Things, a large number of fast operations are required, and the efficient operation mode brought by cloud computing can provide a good application foundation for it. The development of the Internet of Things promotes the progress of cloud computing technology. However, it is worth noting that scale is the prerequisite for the combination of cloud computing and IoT. Only when the Internet of Things is big enough will it be possible to combine it with cloud computing. However, for small-scale fields, the cloud platform of the Internet of Things cannot be well used. How to make the cloud platform of the Internet of Things still be able to be applied on a smaller scale is the focus of our next research.

## Figures and Tables

**Table 1:** Comparison table of different calculation methods.

Performance index	HTTP	MQTT	EDP
Confidentiality	Yes	Yes	Yes
Economy	No	Yes	Yes
Stability	No	Yes	Yes
Dissipativeness	No	Yes	Yes
Universality	No	Yes	No
Generalization performance	No	Yes	Yes
Platform sex	No	Yes	No
Firewall	No	Yes	Yes

**Table 2:** Function assignment table of system control quantity.

Fuzzy sets	PB	PM	PS	ZO	NS	NM	NB
−3	—	—	—	—	—	0.5	1.0
−2	—	—	—	—	0.5	1.0	0.5
−1	—	—	—	0.5	1.0	0.5	—
0	—	—	0.5	1.0	0.5	—	—
1	—	0.5	1.0	0.5	—	—	—
2	0.5	1.0	0.5	—	—	—	—
3	1.0	0.5	—	—	—	—	—

**Table 3:** Statistical table of evaluation indicators.

Overall classification	Organization chart	Specific division	Assess
Sports publicity A	Organization	Relevant management team	Qualitative
	Propagandizing	Strengthen publicity work	Qualitative

Sports research B	Research program	Carry out academic activities	Qualitative
	Scientific research	Improve relevant systems	Ration

Physical activity test C	Sport system	Strengthening physical testing	Ration
	Conditions to ensure	Buy related sports equipment	Ration

Physical class D	Physical education	Relevant teaching evaluation	Ration
	Teaching condition	Strengthen the ranks of teachers	Qualitative

## Data Availability

The data used to support the findings of this study are available from the corresponding author upon request.
